# Incredibly emotional: interpreting trustworthiness in Danish courtrooms

**DOI:** 10.3389/fsoc.2024.1457424

**Published:** 2024-12-16

**Authors:** Louise Victoria Johansen

**Affiliations:** Faculty of Law, University of Copenhagen, Copenhagen, Denmark

**Keywords:** legal decision making, courtroom ethnography, law and emotion, intersectionality, cultural norms

## Abstract

This paper explores how Danish legal professionals assess the trustworthiness of victims in criminal cases based on emotional expressions. It focuses on the alignment of these expressions with the nature of the crime, the social context, and the victims’ social identities, and is based on findings from several ethnographic projects involving extensive observations of crime cases and interviews with criminal justice professionals. The research analyzes how victims’ emotional expressions are scrutinized and interpreted within the context of Danish cultural norms, which favor “calm and quiet” behavior. Legal professionals define this behavior as specifically “Danish,” and often contrast it to ethnic minorities’ way of enacting emotions. Emotions are thus culturally and socially interpreted in courtroom settings, and I relate these findings to broader discussions about how emotions mediate, co-create and maintain systematic differences based on gender and ethnicity in legal decision-making. The study thus highlights the cultural and social dimensions of emotions in this legal setting and calls for greater awareness of how these factors influence the assessment of trustworthiness.

## Introduction

Evaluating trustworthiness constitutes a central yet difficult to articulate aspect of any criminal court case. Nevertheless, prosecutors and defence lawyers continuously negotiate the credibility of victims and defendants, while judges, for their part, often refer to trustworthiness in the judgments they deliver. This paper examines how legal professionals evaluate credibility based on the extent to which they perceive victims’ emotional expressions to be aligned with the nature of the crime, the persons involved in it, the social environment in which it took place, etc. It specifically argues that the nature of the crime *and* the victim’s social identities play a significant role in determining this credibility assessment based on emotions.^1^ In doing so, it contributes to the growing interest in how victims are perceived in court, shifting some of the scholarly attention that has traditionally focused on defendants’ emotional expressions during legal proceedings (see, for instance, Field and Tata, 2023).

Based on five different ethnographic projects conducted in Danish criminal courts, and observations of 63 cases of minor and aggravated violence as well as interviews with 102 legal professionals such as judges, prosecutors, victim’s councils and defence lawyers, I analyse how Danish legal professionals expect victims to feel “appropriately” during trials. Interestingly, the Danish Institute for Human Rights recently found that victims with ethnic minority backgrounds more often experience the acquittal of their perpetrator than ethnic majority victims. It is not my intention, however, to link judges’ impressions and statements with conviction rates or specific sentencing outcomes in each case, but to show a range of possible interpretations of the emotions and behaviors that victims display in a court setting. These interpretations are shaped by specific “emotion cultures” in crime cases which regulate what the victim “ought to be feeling” (Bandes, 2009, p. 7). These feeling rules are not only played out by virtue of the legal professional context but presumably also because of a legal and cultural tradition in which Danish emotional behavior is culturally understood as “calm and quiet” (Johansen et al., 2023). Legal professionals’ perception of displayed emotions in the courtroom contributes to their understanding of cases and victims, but this understanding may itself be rooted in specific majority experiences and cultural self-descriptions. I show how prosecutors seek to translate the “incomprehensible” feelings of victims from ethnic minority or socially deprived backgrounds to the judiciary, while judges, for their part, are concerned with classifying and decoding the expressed emotions according to their own social and cultural knowledge.

The analysis thus seeks to merge several different but related theoretical approaches, drawing on emotion theory, cognitive anthropology, and intersectionality theory. Since emotions encode significant social information, they constitute a critical link in cultural interpretations of action and are likely to be actively used in the negotiation of social reality. Cognitive anthropology provides a useful framework for analysing how cultural understandings and meanings are constructed, while intersectionality theory can highlight the multiple identities at stake in the courtroom through the interweaving of different social categories.

## Research on emotion and credibility in law

This subsection will outline key perspectives on how emotions influence the evaluation of trustworthiness in the courtroom, and is centered on the temporality of emotions, biased empathy, and gendered and racialized emotions in the courtroom.

The temporal perspective is pivotal for analysing emotions in criminal court cases. Firstly, victims’ and defendants’ emotional expressions are often recorded from the moment the crime is reported, usually by the police. The parties’ immediate reactions to the crime are documented and constitute knowledge about them that is put into the case file that judges will receive prior to the court hearing (Johansen et al., 2023). Secondly, the parties in the courtroom are often asked to recount their experiences and emotions before, during and after the offence (Scheffer, 2010). This temporality of emotions is used to understand the emotional reactions of those involved and to place them in time and space (Johansen, 2023). Bladini et al. (2023), for instance, describe how legal professionals strive to present and explain their party’s emotions before, during and after the criminal event as being normal, understandable or necessary, and conversely to present the opposite party’s emotional reactions as incomprehensible, out of line etc. The legal professionals thus receive and consider information about the parties’ emotions along the entire course of the case, which affects their judgment of lay people’s credibility (Ellison and Munro, 2009; Field and Tata, 2023).

A third way in which the parties’ emotions play a role in the courtroom relates to the actions in court as they unfold *during* the trial. This may be in the shape of anger directed at another witness or the prosecutor, or the fact that lay persons may not wish to or are unable to react as the court expects, and generally perform either too little or too much emotion (Rose et al., 2006). The behavior of the parties in the courtroom therefore often requires “translation,” especially by the prosecutor and defence counsel (Flower, 2019; Törnqvist, 2022). This translation is driven by legal professionals’ ability to demonstrate professional empathy, i.e., to try to understand the actions, feelings and motivations of others (Bandes and Blumenthal, 2012; Bergman Blix, 2019). In this way, empathy is used to manage the courtroom and any outbursts of emotion from the parties (Wettergren and Bergman Blix, 2016; Flower, 2019), as well as to elicit information and convey knowledge about the case and its parties (Rossmanith et al., 2018).

Christie’s (1986) noted that victimhood is not objectively assessed but rather evaluated on a continuum of “idealness,” but he did not explicitly consider what might constitute a victim’s ideal *emotions* (Bosma et al., 2018). Victims are generally met with emotional expectations of being able to constrain their possible anger (Miers, 1990; Van Dijk, 2009), just as the specific performance of anger in the courtroom is sanctioned by the emotional regime of the criminal trial (see, for instance, Bosma et al., 2018; Rose et al., 2006; Schuster and Propen, 2010). It may influence the judiciary’s impression of the victim if the victim does not react according to these expectations, affecting their sympathy toward these victims (*cf.* Törnqvist, 2022), and consequently, jeopardizing the victim’s trustworthiness. These legal feeling rules are interactive and relational (Williams, 2009) in the sense that their meaning shifts according to social situations and expectations within specific institutions and are dependent on broader cultural values (Bandes, 2009; Minissale, 2023). They are also unevenly distributed, because gender, ethnicity and social class come into play and co-define the permissible emotions in the courtroom (Weenink, 2009). This interconnectedness between the “art” of empathy and the ability to translate the emotions of others into the courtroom context is therefore challenged by the fact that it may seem easier to empathize with and thus understand the emotions of the parties if they are “similar others” racially and demographically (Manne, 2017; Lynch and Haney, 2011). For instance, ethnic minorities may be described as reacting in specific ways according to their “culture” and are more easily dismissed as untrustworthy (Baillot et al., 2014; Johansen, 2019; van Oorschot, 2020). Legal professionals’ evaluation of trustworthiness rests on their ability to translate information about people and events according to their own prior experiences and cultural knowledge that go far beyond the legal setting (Johansen et al., 2023).

Analysing legal professionals’ own epistemic emotions (such as contempt, disgust, anger etc.) which they bring into the courtroom or feel during the case further deepens our understanding of the interplay between lay and legal parties and how emotions are co-constructed through this interaction (Anleu and Mack, 2023; Törnqvist and Wettergren, 2023), but lies beyond the scope of this article. Conversely, focusing only on legal professionals’ *own* emotions may lead us to overlook the ways in which they categorize and interpret lay people’s emotions according to assumptions about own and other “cultures.” This is why I focus on the cultural conditions for the “decoding” of emotions in the analysis. The aim of the present study is thus to contribute to the well-established research about how legal professionals’ own emotions shape courtroom interactions and judgments by focusing on the issue of how *perceptions* of others’ feelings influence evaluations of trustworthiness in court.

## Cases and courtrooms

### The Danish criminal legal system

Scandinavian law has been characterized as a separate legal system (Tamm, 2008), and as a system adhering to civil law, but with elements of common law procedure particularly concerning its adversarial mode (Anderson, 1992). Denmark does not have specialized investigating judges, and the Danish system of criminal and civil courts at the trial stage is simple and unitary. The prosecutor must be objective during the trial, and is required to provide information that is relevant to the defendant’s guilt as well as information that speaks toward their innocence. Judges’ assessment of evidence is free, and the judgment is based solely on the evidence presented at the hearing in court. The degree of proof is not codified, but certainty beyond reasonable doubt is settled practice, however, the defendant has a “burden of explanation” (Langsted et al., 2019).

Denmark has 24 district courts processing both civil and criminal cases. They form part of a three-level court system consisting also of a Higher and a Supreme Court. At the district court level, which constituted my level of inquiry, the prosecutor initiates the criminal proceedings in the courtroom by reading aloud the indictment. Afterwards, the defendant states whether they plead guilty or not. The defendant is then interrogated—first by the prosecutor and then by the defence counsel. The defendant is not obliged to make statements or answer questions, due to the right to be silent. Witnesses are then brought into the courtroom—victims first—and usually interrogated first by the prosecutor and then by the defence counsel. Victim impact statements are not accepted, at least formally. Usually, the prosecutor brings the victim and other witnesses into the courtroom. Victims of violence are offered free legal representation to assist them throughout the criminal justice process. The counsel’s role includes supporting the victim during interrogations and court appearances, explaining legal proceedings, and aiding in claiming compensation for damages. The interviewed counsels described their role in violence cases as preparing victims for court and guiding them on how to communicate and act during the trial. However, not all victims, especially in less severe cases, utilize this opportunity. The present studies therefore do not address the variation in the appointment or role of counsel based on factors like gender or ethnicity since not that many counsels were appointed. If a victim’s counsel has been appointed to the case, they will enter and leave the courtroom together with the victim(s). During the interrogation, which is always performed with the defendant or witness sitting at a desk in the middle of the room facing the judges, the counsel will usually sit next to the victim at the desk. In cases where victims do not have counsel, the prosecutor often takes on some of the responsibilities toward the victim.

If there are documents relevant to the case, such as a medical certificate or a criminal record, the prosecutor reads them aloud after all witnesses have testified. Finally, the prosecutor and defence counsel put forth their closing remarks in the case, discussing the question of guilt as well as proposing a sentence. At the end of the proceedings, the defendant has the opportunity to make a statement. The judges then leave the courtroom to deliberate. In the cases of violence I observed, where defendants had pled not guilty, and with a maximum penalty range of 4 years, a career judge and two lay judges would always participate. They have one vote each concerning issues of guilt as well as sentencing.

### Methods and data

I have been collecting data for more than 15 years within this legal framework and involving courtroom ethnography in five different projects. The analysis in this article is based on these research projects in Danish court cases from 2007 until now. Not all of them aimed at studying victims and the way they are understood and treated in the courtroom, but my field notes and records from the courtrooms have naturally involved all professionals as well as victims and witnesses. There are several constants across the projects that make them suitable for inclusion within an overall analysis. Firstly, all projects have focused on simple and qualified violence, i.e., within the framework of offences against the person. The projects were based on qualitative methods in the shape of observations of criminal cases in the courtroom, just as they interviewed legal practitioners and their experiences of the cases. During these trials, field notes were taken of the judges’ gender, age group, appearance and participatory role in the trial, including guidance to defendants, victims and witnesses, as well as (if any) reprimand of the parties, and how this was communicated both overtly and indirectly. Similarly, the appearance of defendants, witnesses and spectators was noted, as well as the treatment of defendants, witnesses and audience in terms of credibility, the way in which they are addressed, etc., by judges, defence counsel and prosecutors. Two of the projects have also had a processual perspective, in the sense that they have focused on how credibility and impressions of both defendant and victim are co-constructed along the entire criminal process from reporting at the police to legal decision-making (Johansen, 2015; Johansen et al., 2023). The purpose is to synthesize qualitative findings across this related, ethnographic research, identifying common themes, analogies, key metaphors, etc., but I do not strive to aggregate data (*cf.* Noblit and Hare, 1988).

I present the premises and purposes behind two of the projects from which I have chosen examples and citations, although my other projects serve as a contextualization of the data and findings in the paper. My PhD project 2008–2012 explored in broader terms what significance the defendant’s “personal circumstances” as collected by the Prison and Probation Service and presented in the courtroom—and used during the deliberations—could have on the legal practitioners’ understanding of the case, their involvement in the defendant and on the decision itself (Johansen, 2015). I observed 32 cases in court, and participated during judges’ deliberation. After the court cases, the judges, defence lawyers and prosecutors in question were interviewed about their impressions of the particular cases, defendants, victims, witnesses, pre-sentence reports, etc. A total of 38 criminal justice actors were interviewed for this project.^2^

Finally, together with colleagues (2017–2020), I investigated victims of violence and their way through the criminal justice system from reporting and until the case was closed (Holmberg et al., 2021). Part of this project studied the ways in which the professional actors interpreted and classified victims’ emotional reactions based on both an overarching, legal institutional understanding, as well as on the basis of their specific roles as either police, prosecutors, judges, etc. (Johansen et al., 2023). We conducted 120 interviews with 59 victims, 37 legal professionals (judges, prosecutors, and defence lawyers), observations of 26 initial interrogations with the police, and 14 court cases.

All respondents across the projects have received information about the projects, how the data would be used, as well as their right to withdraw their participation in the project at any point. Data has been securely stored according to the guidelines of the Danish data protection authority, and all respondents have been anonymized, as well as identifiable details of the criminal cases in question.

The empirical examples in the analysis are chosen based on their general occurrence, implying that I have not chosen “outliers.” I have also striven to use data from both projects, and from different cases and informants involved.

## Culture, cognition, emotion: an analytical approach

In accordance with my theoretical choices as mentioned above, the subsequent analysis of my data focuses on eliciting the conceptual models that legal professionals use in this legal setting, rather than focusing on a description of their own behavior (Boster, 2011).

Although there is a general agreement that emotions form an integral part of decision-making in law (Bandes and Blumenthal, 2012), there is less consensus as to the boundaries or even connections between concepts such as emotions, language, body and cognition (Finkel and Parrott, 2006). For instance, Bergman Blix and Wettergren (2018) use the concept of an emotive-cognitive judicial frame that shapes legal professionals’ perceptions and performance of their work as being predominantly rational. What I am referrring to in the following, however, are two theoretical traditions on cognition and emotion that originate outside the legal realm.

This article defines cognition as the way in which people process ideas, impressions, emotions, etc. through the relationship between individual and culture (Mukhopahay, 2011). My use of cognitive anthropology as one of several analytical approaches should therefore be understood to encompass the role of situated bodily practice in the courtroom and how people process and interpret their impressions of others’ emotions (*cf.* Zajonc, 1980; Kronenfeld, 1996). Cognitive anthropology has dealt with issues such as the importance of emotions for both thought (Rosaldo, 1989) and decision-making (Gigerenzer, 2007), drawing on cognitive psychology in order to explain the relationship between culture and person (Holland and Quinn, 1987).

Although early cognitive anthropology—and cognitive theory in general—has been associated with thought and rationality and criticized for being reductionist because of its metascientific basis, behind the designation of the “cognitive” research field there is a wealth of diverse and complex research addressing a number of topics related to human consciousness such as learning, evaluation, emotions, motives, intentions, etc. As Anderson (2011) describes, researchers have also been actively engaged in integrating the field of cognition into emotion research since the 1950s, underscoring for instance how emotions and their cultural contexts influence the processing of social judgments (Forgas et al., 2003). In this research, the focus has been on the fact that people’s cognitive engagement with their surroundings is context-dependent, and includes both sensation and intellection (Ellen, 2011).

Within cognitive psychology, prototype theory has shown that humans use categories to gather knowledge quickly and make decisions based on this information (Rosch’s, 1978). Within a category, some examples are more illustrative than others and are called “prototypes” (*ibid.*). People’s decisions rely on these distinctions between ideal and more peripheral examples, which in turn means that they rest on biases and preconceptions which allow them to be made quickly. Gigerenzer (2007) refers to this phenomenon as the sacrifice of accuracy in favor of efficiency, noting that it can be a necessity in many situations and may even fundamentally ensure survival. Forgas et al. (2003) links these responses to emotions, stating that we also categorize by using emotions, and that these emotional responses to a situation, a person or an object can be produced in a fast and automatic way. These perspectives are relevant for the legal practitioners in the courtroom, who must make decisions based on impressions gathered within a short timeframe. According to Lakoff (1987), when exposed to others’ emotions, we use categories to make sense of them based on some joint cultural knowledge. Researchers have used terms such as cultural scripts, schemas, or models to describe these relations between self and society (e.g., Ewick and Silbey, 1998; Holland and Quinn, 1987; Sperber, 1996), of which I will use the terms models or schemas in the following. These are defined as joint experiences connected with the system of values shared by the majority of members of a certain ethnic or social culture, and they offer flexible templates for understanding a given situation or event in the sense that the interpretation of new situations is shaped by interpretations of past experiences (Ewick and Silbey, 1998).

Although research into cognition and emotions cannot be fully separated, the research traditions are nevertheless different. Some similarities include research showing that emotions are collective, historical and cultural phenomena (Williams, 2009), and may thus be studied as embodied thoughts (Nussbaum, 1996; Rosaldo, 1984). Differences between the theoretical approaches include the fact that emotion theory often analyses the role of emotions in relation not only to knowledge production (like cognitive theory), but also to the production of subjectivities (Harding and Pribram, 2009; Jaggar, 1989). Within sociology, studies of emotions have shown how they are socially construced and imbued with feeling rules and emotion management (Hochschild, 1983). Situating emotion reserach within the legal realm, research has discussed the role of emotions in sentencing and decision-making processes. For instance, Bandes and Blumenthal (2012) state that emotions are a set of evaluative and motivational processes that help categorize and interpret information, and influences how we evaluate the intensions and credibility of others, constituting dynamic processes integral to decision-making.

While the field of law and emotion thus explores the relationship between emotions, cultural expectations and decisions, emotion research has generally been criticized for overlooking the role of social positioning (Harding and Pribram, 2009). Emotional research that is situated within culture studies emphasizes topics such as power relations, and views emotions as communicative, intercorporeal, and intersubjective. The expression and perception of emotions are viewed as the convergence of physical, linguistic, and distinctly sociocultural dimensions (Harding and Pribram, 2009). They should be seen as part of power relations between people, rather than as individual subjectivities (Burkitt, 1999; Williams, 2009), and as an embodied emotional experience that is created relationally and interactively. This approach to emotion thus bridges the field of law and emotion with that of intersectionality.

Intersectionality theory probing into junctions between age, gender, ethnicity, and class as formulated by Crenshaw (1989, 1991) and Collins (2009), can help to bring historical and political perspective to the study of emotion, e.g., in relation to the marginalization of, for example, women and people of color, who have often been considered “emotional” in a specific national and cultural context in the sense of being irrational, subjective etc. (Fricker, 2007). Intersectionality theorists highlight and recognize the multiple identities at stake in social situations through the interweaving of different categories (Collins, 2009; Lutz et al., 2011), which is highly relevant for understanding the ways in which legal professionals assess some victims as more trustworthy than others based on their displayed emotions. Bonilla-Silva (2019) describes how the category race is produced through and with emotions, because people experience and interpret racialized relationships emotionally (see also Denzin, 1984). Bonilla-Silva further argues that the emotions of the dominant race become authoritative since they are seen as the “correct” way of feeling, a point that situates the Danish feeling rule “calm and quiet” within a hierarchy of emotions connected to broader structural differences. This helps us shift attention from a definition of emotions as something people have or enact at the microlevel as part of their identities in courts or other settings to what emotions *do*, and how they as such are deeply rooted in broader structural productions of difference (Ahmed, 2004; Fricker, 2007). The intersectional dimensions of emotion are thus linked to a politics of inequality (McCall and Orloff, 2017). People are generally expected to express emotions in ways that align with their category membership: emotional expectations for women differ from those for men (Collier, 1998; Jaggar, 1989; Lutz, 1990), and ethnic minorities are often characterized as responding in specific ways based on their perceived “culture” (Baillot et al., 2014; Harding and Pribram, 2009). For instance, Fischer (2000) and Shields (2002) discuss how emotion plays a crucial role in constructing concepts of femininity and masculinity, such as the belief that women are more likely to express emotions like sadness, while emotions like anger are considered less acceptable for women to display. Furthermore, categories are constructed relationally in the sense that it may carry quite different meanings entering the courtroom as a white woman or a woman of color, or as a young woman versus as a middle-aged woman (*cf.* Bonilla-Silva, 2019; Harding and Pribram, 2009; Hooks, 1989).

In summation, cognitive anthropology offers a framework for analyzing how cultural understandings are constructed with and through emotions, while intersectionality theory sheds light on the multiple identities at play in the courtroom by examining the interconnections between various social categories. The social construction of emotions, viewed as cognitive evaluations that consider multiple subjectivities and cultural contexts, highlights how these dynamics shape legal professionals’ perceptions and interpretations.

## Analysis

The analysis takes its point of departure in a generalized understanding of different cases in which violence was perpetrated, and the accepted emotions that professionals link to them. I first introduce the broader Danish feeling rules that may help contextualize the legal settings and their interactions. This part focuses on “the ideal victim.” The analysis then proceeds to show how these feeling rules are challenged by the perception of different “kinds” of victims depending on their gender, social class and ethnicity. Using an intersectional lens, I show how legal professionals classify the type of victim they associate with in the courtroom, and the credibility they believe can be deduced from different categories of victims’ emotions. The analysis focuses on how victims are supposed to behave emotionally *in* court, and thus delimits itself from analysing victim emotions from a range of other temporal perspectives including expected emotions at the time of the crime.

### “Calm and quiet” as a Danish feeling rule

This section describes a broader cultural context, in which criminal justice actors generally defined a “Danish” way of acting in court as “calm and quiet” and in opposition to, for instance, ethnic minorities’ way of acting (Johansen et al., 2023). The intention is to analyse terms such as “calm and quiet” as cultural models or schemas, i.e., as expressions of the pragmatic interaction between individuals who communicate using public representations (Sperber, 1996). Ewick and Silbey (1998) use the concept of cultural schema to describe these relations between self and society. Cultural schemas offer flexible templates for understanding a given situation or event in the sense that the interpretation of new situations is shaped by interpretations of past experiences. However, it is not my intention to present understandings such as “calm and quiet” as culturally unique Danish understandings of appropriate emotions in specific institutional contexts such as courts (*cf.* Anderson, 2011), but to explore what it means for professional actors themselves that they meaningfully apply this understanding to assess victims’ (credible) responses. Rather than treating “calm and quiet” as a cultural essence, I see it as an emotional expression of an ongoing construction of powerful majority relations at the expense of other identities in the courtroom and beyond (*cf.* Bonilla-Silva, 2019). Throughout the analysis, the notion serves a dual purpose: first, as a reflection of Danish legal and social values, functioning as a cultural descriptor; and second, as an analytical lens through which I study how victim performances in court are challenged by legal professionals based on this cultural self-perception.

A common thread surfacing in the empirical material from the different projects has been criminal justice actors’ perception that some reactions from both defendants and victims could seem either too downplayed or too exaggerated, and that this seemed out of place both from the standpoint of a legal norm and from a broader understanding of Danish culture.^3^ The meaning of this is analyzed in the following primarily on the basis of statements about victims, and observations from the courtroom. In the following examples (A-D) from interviews with judges and prosecutors, “calm and quiet” is used as a recurring, stable, linguistic phrase:

A. Prosecutor Sofia: I also think that they give an explanation quietly and…

Prosecutor Erik: credibly…

Prosecutor Sofia: If you feel they are affected, then it is also okay for them to be so. So an explanation… A victim who comes in calmly and quietly – you can be a little ill at ease, that does not matter – but it…. Just the fact that it’s calm and quiet and you get the explanation, right? (Group interview 2019).

B. Judge Camilla: But I also want to say that I agree with Emilie that, at least in my experience as a judge, that most of the time it works calmly and quietly for everyone. (Group interview 2019).

C. Prosecutor Kirsten: When I saw the witness in court, he was a nice guy who sat calmly and quietly… (Interview, 2010).

D. Judge Lene: I think many of those who give evidence here give a calm and quiet explanation. Ehm but. But these are also common cases of violence. I do not remember having had an aggravated case of violence recently, at least. It may have been the case, but right now I cannot think of any. So it’s ordinary everyday violence. (Interview, 2019).

There seems to be both a linguistic, a bodily and a spatial dimension to the term “calm and quiet.” In example A, the victim enters the courtroom “calmly and quietly.” Thus, the victim’s physical actions in the room are taken into account. Examples C and D express ways in which the victim gives evidence, which, according to the actors, is predominantly calm and quiet. Moreover, in example B, the judges characterize the entire trial as being generally “calm and quiet for everyone.” This denotes that both the case as an overall event and the parties involved in it are calm and quiet. As described in our previous study on victims (Johansen et al., 2023), the phrase “calm and quiet” was mentioned 27 times in 37 interviews with professional actors. It may seem very straightforward that the professional actors highlight—and prefer—a calm and quiet performance and explanation from the victim (and others). But “calm and quiet” might actually connote values both within the Danish legal system and more generally, just as it may serve to include as well as exclude certain kinds of victims. One way of showing what the concept connotes is by distinguishing its immediate opposite as expressed in the data. In a group interview, prosecutors expressed that it made their job easier if the victim had a certain behavior:

Prosecutor Nanna: Who puts it forth calmly and quietly and… Yes, seems credible in the way they say it.

Prosecutor Erik: And yes, just not overdramatizing or… there is… Sometimes it also really gets so inflated. Just quietly, soberly, right? (Group interview 2019)

In this quote, a quiet explanation is contrasted with an overdramatization, an exaggeration, just as it is associated with “sobriety,” which, all other things being equal, connotes “credibility.” It’s precisely credibility that can be jeopardized by an overreaction, as stated by these prosecutors:

Prosecutor Ingrid: But the aggrieved people who kind of uh overreact and stuff like that, they're kind of hard because you have to kind of explain to the judges why… Because it seems weird doesn't it? That it's not just for the fun of it that we're there and

Prosecutor Diana: …A dog and pony show

Prosecutor Ingrid: to explain, is it fantasy we’re listening to, or is it real? (Group interview 2019)

According to these prosecutors, they must come up with an explanation when dealing with victims who react so strongly that judges may find the emotional reaction incomprehensible, or verging on incredible.

At first glance, the phrase “calm and quiet” seems like a distinctive way of handling emotions, and, by extension, as a way of characterizing (un)desirable emotions. Research has shown that this “value” may also be found in other institutional settings in Denmark such as in schools (Gilliam, 2014). I identify it as a cultural model, defined as joint experience connected with the system of values shared by the majority of the members of a certain ethnic or social culture (Sperber, 1996). Rosaldo (1984) shows, for instance, how social groups define themselves as “selves” through specific emotions, and that this therefore creates specific attitudes toward emotions. Theories of intersectionality, however, contribute to this understanding by elucidating how the phrase not only structures how emotional expressions are handled and interpreted in the courtroom or other contexts in Denmark, but also classifies undesired emotions along social and cultural divides (Harding and Pribram, 2009). “Calm and quiet” should thus be contextualized as an ethnic Danish, middle class model. The fact that other cultures and countries might share this idea of “calm and quiet” does not necessarily undermine its power to name and exclude in a Danish setting (*cf.* Ewick and Silbey, 1998).

### Different kinds of cases and emotions

As shown in the previous section, victims’ emotions are carefully monitored and evaluated in the courtroom according to broader understandings of appropriateness. I now proceed to map the ways in which *kinds* of cases, defendants and victims elicit different expectations vis - à - vis their reactions in the courtroom. The first distinction concerns offences against the person versus cases of fraud or theft, which was recurrently addressed by legal professionals in interviews, for instance by this prosecutor:

Prosecutor Diana: I think it's quite natural that when it's something dangerous, you're present in a completely different way. You ask in a different way than if it's burglary, or I don't know what else it should be… something more material. Fraud, one's account that has been blocked, or a bike that has been stolen… I think you use your empathy and– at least when it's serious.

The quote corroborates that the kinds of cases in which one can be made a victim vary in nature. Dangerous crime leads legal practitioners to act differently toward victims, and several judges and prosecutors mention empathy in their approach to said victims. In addition, psychological experiments show that people expect different intensity in victims’ emotional responses according to what they have experienced (Rose et al., 2006). What I want to develop further here is the suggestion that some types of crime make specific professional understandings of the victim possible (Törnqvist, 2022; Wallin et al., 2021). Violence as a subcategory of crime sets the stage for certain emotional victim responses, depending on their victim status. If we look into this subcategory, the legal professionals further perceived differences within it which evoked an emotional dimension, as Judge Minna explains:

Judge Minna: Yes, and then just the nature of the case, right. If it is someone who is on their way home alone and who has suddenly been attacked, it is a completely different situation you are in than if you had been arguing with one of your good friends, who you just got mad at in the queue down at the bar. It's a completely different situation then.

Interviewer: Can you elaborate on how that might be?

Judge Minna: Well, it is clear that… that for most people it is somewhat more traumatizing to be attacked on eh… when one walks unsuspectingly down the road, rather than when being part of a quarrel that develops between two more or less equal parties. (Group interview 2019)

This quote, in line with many other interviews, distinguishing between violence in private life, random street violence, and violence in the context of nightlife, reflects Christie’s (1986) definition of “ideal” victims as innocent, unsuspecting, with no prior “history” with the defendant, etc. It also reflects a general view among legal professionals about certain victim-offender overlaps. What Christie may have overlooked, however, is how the ideal victim can express emotions that other “kinds” of victims may not (Bosma et al., 2018; Johansen et al., 2023), i.e., the right to feel something specific depends on a combination of the type of case, the victim’s role in the violent episode, and the victim’s other social characteristics.

As expressed in the last quote, the judge expects that the more “unsuspecting” the victim, the more they will be traumatized from the violence. It also suggests that the person would be expected to emotionally process this violent experience in other ways if he or she had played a different role in it. For example, among the judges we interviewed in 2019, some mentioned that victims can also sometimes contribute to fuelling a conflict:

Judge Karen: There are also many who feel ashamed and find it embarrassing that they have gotten into this situation and who are, well… Again, there are always two sides to an issue, right? After all, it's… It may well be that there are some things they think are not so funny - or are not so nice to tell - it is rarely the *very very* sweet, *very very* young… (The other judges laugh)

Here some slightly different emotions are at stake. The judge references being embarrassed, feeling ashamed, and that as a victim you may not find it particularly amusing to recount the incident in the courtroom. This, of course, first and foremost suggests a form of “complicity” as expressed in the judge’s concluding remark, in cases of violence you rarely meet very nice, very young victims, which can also be seen to imply very innocent or sympathetic victims. Where the first characterization of the unsuspecting victim suggests something traumatizing, the other feelings described are in a way more sordid. Being ashamed and embarrassed is qualitatively different from being traumatized by an incident of violence.

Although the quotes reflect the often-cited notion of the ideal victim, they categorize victimhood in relation to a particular type of violence case, based on a classification requiring prior knowledge of both. The quotes reflect the emotions that different kinds of victims might be expected to exhibit in the situation, whereas the display of other emotions would seem untrustworthy. The innocent victim will probably not feel embarrassment, and the complicit victim cannot credibly express trauma. They are both socially limited if they are to express an emotion that the judges find comprehensible.

In order to further develop the idea of the “ideal” victim, I will therefore return to Rosch’s (1978) prototype theory. Rosch pointed out that when people categorize an everyday object or experience, they rely less on abstract definitions of categories and far more on a comparison of the given object or experience with what they consider to be the object or experience that best represents a category. There is a graded degree of belonging to a conceptual category, and some members are more central than others. Prototypes and gradations lead to an understanding of category membership not as an all-or-nothing sum, but as more of a web of interlocking and overlapping categories. The prototypical features of the victim category emerge from examples of cases of violence (in private life versus “gratuitous” street violence, for example). They were also expressed linguistically in how prosecutors and judges talked about victims depending on whether they were more or less “exemplary.” The prosecutors mentioned a “criminal victim, an “impartial victim, a “certain clientele,” “a victim that is actually a victim,” “where the victim is not a real victim at all,” etc. Categorizing in the way that legal practitioners do draws on broader assumptions about the victim. This is due to the fact that a set of associated meanings and values is activated which surpass the category of “victim” itself. When talking about “a certain clientele,” for instance, prosecutors are also indirectly suggesting that this may be a group of people used to being involved in crime, whether as victims or defendants. For example, one prosecutor says that she does not make much of an effort to advise certain victims about the process of the criminal proceedings because they already know it well:

Prosecutor Sofie: Because these practical questions about how it works, at least a certain clientele knows how it happens down in court, and then they may still be nervous about having to face their attacker, but at least they physically know where they’re going; and who picks them up (in front of the courtroom) and so on. (Group interview 2019)

The physical confidentiality, or lack thereof, is thus in itself a sign of the person’s impartiality and innocence. Earlier analysis in the field has investigated how the perception of a “criminal victim” can cause prosecutors not to take as much care of the victim (Johansen, 2015; Johansen et al., 2023). Prosecutors’ self-perception as being professionally empathetic is thus contested by their more or less implicit categorization of “unworthy” victims.

Like prosecutors, judges also tried to get an overview of the victim and the case by trying to classify them through the attribution of different kinds of victim status. For instance, a number of judges stated that it could be uncomfortable to testify in court “if the person is really a victim,” which suggests other options for not being a victim. Examples of the latter emerged through statements such as “the alleged victim in question,” “allegedly aggrieved,” “who was actually the victim,” “a possible aggrieved,” “who is allegedly wronged here,” “the one who claims to be the victim,” etc. These phrases communicate objectivity as well as (im)partiality. As stated in a previous article (Johansen et al., 2023), they related to the judges’ self-perception as members of a profession that must objectively decide on a case and therefore cannot reach a verdict on any person’s “legitimate victimhood” until the very end of the judgment. Nevertheless, the following quote suggests that some (violent) emotions may be more justified if the victim’s “status” within that category is taken into account:

Judge Berit: There may also be someone who is actually seriously aggrieved and who is quite aggressive and annoyed by what has happened, right.

One might tentatively point out that, in the eyes of this judge, the victims who are “seriously aggrieved” may be somewhat more justified in exhibiting emotions such as anger or aggression. The judge does not elaborate on what she means by “serious,” but it may be understood as an opposition to the otherwise common assumptions that some victims bring the situation on themselves, or have been part of the conflict, or that the case of violence does not seem very serious in itself.

### Different kinds of victims—different kinds of accepted emotions

The previous section has shown how certain kinds of cases and victims are evaluated and judged on the basis of the emotions associated with them, and that legal practitioners similarly expect certain sets of emotions to be expressed by victims depending on their role in the violent episode. In this section, I explore these differences in more detail. Intersectionality theory is used to highlight the multiple identities at stake in the courtroom as social categories such as class, gender and ethnicity interact.

I will begin by looking at an example from a court case, showing how a woman victim of violence was understood through a combination of her explanations and feelings, which were expressed linguistically as well as physically. Theories about emotions have been criticized for not taking embodied social positioning into account (Harding and Pribram, 2009). Conversely, intersectional theories have focused on structural inequality and generally underplayed how social categories are also constituted through emotions (Lutz, 1990). The following example illustrates these different dimensions of embodied cultural knowledge:

I am at the police station when victim Sandra comes to report some blows that her neighbor Rie has given her. The violence is the culmination of years of harassment, where, according to Sandra, the neighbour has thrown garbage in her garden, stalked her, cut open her car tires, etc. Sandra seems scared, and she does not dare to go outside her home. The police officer is very responsive to her and notes down. He also urges her to seek a restraining order. After a few months, the case goes to court, where I meet Sandra again. She must give evidence. Both Sandra and Rie are between 40 and 50 years old, Sandra a little older. Both are ethnic Danish. The prosecutor shows Sandra a lot of pictures of her bruises and asks about the blows, was she beaten with the right hand, left hand, etc. […]. Meanwhile, Sandra has become a bit agitated, not angry, but agitated, she speaks loudly, and when she gets questions about the blows, she gets up from the witness stand and shows where she was kicked, etc. She is very physical in her explanation. [I think if you don't know Sandra, you'll think she's a bit aggressive.] The court finds Rie guilty, she receives a 30-day suspended sentence, which is relatively lenient. The judge justifies this by saying that there “has been a conflict” between the two women. After the verdict, everyone leaves the courtroom, except the prosecutor and two attorneys from the Ministry of Justice, who were supposed to provide feedback on the prosecutor's performance. The judge also comes in briefly and talks to the new prosecutor. All three believe that both women probably had it coming. The two clerks say they would have "gone tougher" on both the defendant and victim. They thought Sandra sounded like she herself had been a big part of it, and like a type that could create a conflict. (Field Notes 2019)

My own perception that Sandra may appear aggressive in the courtroom turns out to be echoed by the other legal professionals, implying that my reflections during observation are not objective, but are shaped by our shared cultural schemas for what constitutes acting aggressively (Atkinson and Coffey, 2001; Johansen, 2019). The fact that Sandra appears quite physical, bordering on angry or aggressive, when giving her account is interpreted by the legal professionals as a sign that she herself has been part of the conflict and is thus a kind of “accomplice.” They clearly do not expect this reaction from her, and their response is to suggest going “tough” on her. Her anger is not interpreted as an outburst, justified or otherwise, triggered by years of harassment, but as a sign of a conflict with two equal sides between two women. Sandra’s “emotional purity” as ideal victim is contested by her apparent aggression, implying that emotionally “muddy” cases prompt the legal professionals to take into consideration both sides of the conflict to a greater extent than emotionally trustworthy victims. Constructing this kind of knowledge is thus a collective project since emotions exist in dynamic relationships between lay people, professional structures and specific cultural contexts (*cf.* Bandes, 2009; Kenney, 2004). They are continuously expressed and evaluated in the court’s social interactions between a victim/offender and legal professionals (Anleu and Mack, 2023). The dismissal of Sandra’s outburst can therefore be interpreted both as a consequence of her general status as a victim and as a manifestation of gender and age-based expectations. Victims are generally met with emotional expectations of being able to constrain any aggression they may be feeling (Miers, 1990; Van Dijk, 2009), and the specific performance of anger in the courtroom is sanctioned by the emotional regime of the criminal trial (Bosma et al., 2018; Rose et al., 2006; Schuster and Propen, 2010). A defence lawyer, for instance, stated in an interview that:

“It may make a difference if the defence lawyer can get the victim a bit upset, and maybe make the victim appear a bit like one who has caused something, then they may win over the lay judges and the judge a bit” (Interview, 2019).

This lawyer explains a professional strategy for getting the victim “upset,” and couples anger with issues of complicity and untrustworthiness, which is precisely what happens in Sandra’s case. Additionally, though, Sandra’s case invites reflection on whether different expectations apply differently to women and men. Would her actions have seemed as similarly aggressive if she had been a man? The vast majority of violent cases I have observed included a male defendant, and in about half of the cases, a male victim. It often happens that one or both parties get annoyed or vocal. In one observed case, for instance, a male victim ran out of the courtroom in frustration. The case concerned an escalating street argument at night in Copenhagen. The judge dryly remarked: “an angry young man,” and continued the trial (field notes 2011). No reference was made to a “conflict” between the two parties in the grounds of the judgment. The two examples come across differently in the sense that the “angry young man” fits better into an understanding of who is allowed to express anger as a victim. It is undesirable, but not incomprehensible for the man to express himself like this, and none of the legal professionals subsequently voiced any consternation about that incident in informal conversation.

Women, on the other hand, are expected to express anything but anger (Hochschild, 1983; Lutz, 1990). According to Shields (2002), the question of anger constitutes a fundamental paradox in the prototypical expectation for emotional female/unemotional male. Emotionality is associated with femininity, while anger as a prototypic emotion, is considered masculine. Women are expected to express emotions like sadness or empathy, while emotions like anger are socially restricted for them. Sandra may therefore be affected by the expectations placed on her both as a victim *and* as a woman. She appears more remarkable than the male victim because of the unexpected combination of her raised voice and standing up in the witness stand. This is an example of an embodied emotional concept of anger, in Lakoff (1987) words, which is clearly gendered. It also shows how emotions position individuals within structures of dominance and function as cognitive assessments and moral judgments of the individual’s place in the world (Denzin, 1984; Jaggar, 1989).

Similarly, age is imbued with emotional meanings and positioning (Harding and Pribram, 2009). Within the legal system, there is an assumption that the younger a person is, the more immature and emotionally reactive (Johansen, 2019). Sandra’s triple position as victim, woman, *and* middle-aged may place her reaction in a particularly negative light, since she is possibly also seen as too mature to show anger.

Things may play out differently in relation to sadness as an emotion, as noted by Shields (2002). Several judges stated that young men had fewer opportunities to show how sad and shaken they were after a violent case in which they were victims:

Judge Helle: But a boy who has been beaten by someone, by two or more other boys, has to defend more than just himself. I think he feels, well, I'm a boy, I should be able to handle this myself. There is some pride that is different than if it were a girl who can afford to be sad. (Interview 2019)

Judge Lisa: It is typically men who are subjected to violence in nightlife, so are also more subject to a convention that "If you go out – well, then you may get some blows." So they can't even… So they can't really go in and show how upset… That is, how shaken they are. They just cannot. (Interview 2019)

This attitude was also prevalent among prosecutors, some of whom even believed that men crying in court was “pathetic” (Field notes 2010).

When interviewed about their experience of different kinds of victims, as mentioned, prosecutors distinguished between cases and more or less worthy victims, but also between different types of reactions based on the victim’s social environment, as in the following group interview (2019) with prosecutors:

Sally: Well, it does matter if it's Susanne coming from the abuse environment, who is already piss-drunk when she shows up (in court), etc. And swearing and shouting, versus when old Grethe Jensen comes and has been beaten or has had her bag stolen, or whatever. So that is the ideal victim. That it’s someone who is credible, that you trust her, that she wants to talk…And is affected… I feel like saying…

Kirsten: Yes, it’s okay if they cry a bit.

Here, shouting is considered an inappropriate behavior, and at the same time as associated with a social environment. Both victims are women, but one of them is from an abusive background, which is somewhat akin to prosecutors’ assessment of victims as being from “a certain clientele.” Susanne is not sad, she is angry and noisy, while the older woman is emotionally “affected,” possibly crying. Conversely, a non-reaction from a victim of violence considered “foreign” to risky environments seems equally troubling, as the following example from an interview with a judge exemplifies:

Judge Emma: […] sometimes when you… you may feel surprised that… that someone who is not part of such an environment comes and tells a VERY violent story and can still retain so much control over themselves, then you may sometimes think "hmm?". […] so it forms part of…an assessment of evidence, of course it does, how people they…

Interviewer: it will seem less credible??

Judge: Well, you may find yourself thinking “Oh, can this really be true?” (Interview, 2019).

Here, the victim’s alleged socially healthy environment puts expectations on them to be very distressed about a violent episode that people from “a certain clientele” would be more used to and therefore less prone to be distraught at.

Every time we experience emotions, we use categories to understand and make sense of them (Lakoff, 1987), and in the past examples, intersections between gender, class and age are articulated. This insight can be used and developed to understand not only who behaves most ideally in the category of victim (*cf.* Christie’s, 1986), but also that victims’ different emotions are linked to social markers as purported by intersectionality theory.

The last perspective in the analysis concerns ethnic background. Legal practitioners mentioned that excessive reactions from ethnic minority victims could elicit feelings of untrustworthiness in the court. For example, one prosecutor expressed:

Prosecutor Pia: A lot of the girls I've been down [in court] with, where it's some (break) foreigners, where it's actually victims I'm thinking about now, seem like the cultural differences mean that they're just far out, and they can't quite understand why you're asking them anything. They usually perceive that if you ask them something, even though they have explained it to the police once, and then when I ask again, they perceive it as if I do not believe them, and that is not the case, but they quickly become wronged in their way of acting.

Interviewer: So also the witnesses, the victims?

Pia: Both the witnesses and the defendant I think, and I think that’s their culture, and that they may also feel that they are already feeling bad when they come, and it’s a Danish judge and a Danish prosecutor and Danish defence counsel.

Interviewer: You emphasize the girls?

Pia: I certainly think, especially these fights, that they feel so wronged, and even though they are actually really victims in the case, then…– The specific case that I’m thinking of, well they ruined it for themselves in a way because they were chewing gum, and when I asked them, they responded like, “How would you feel if it were you, do you think?” And if they had told completely objectively how, or not objectively, they are of course allowed to be subjective and be emotional, but how it had happened, and just answered the questions, so yes, it would have had a different outcome.

Interviewer: What was the outcome?

Pia: They were acquitted, it was two guys, also foreigners, who were accused of violence against them, but the girls could not explain at all, yes all wound up […] One of them actually said, “Well, don't you believe me, and why are you laughing?” There was actually a lay judge who sat and laughed or something like that because her behavior was so upset, so completely disproportionate. But of course it was completely wrong that he sits and laughs. It must also be offensive to her. (Interview 2010)

According to this prosecutor, the two victims are somehow responsible for their alleged perpetrators being acquitted, since they did not behave in a way that was emotionally appropriate according to Danish cultural norms, acting disproportionately both in terms of language and aggressive behavior that seems unusual in this legal context (*cf.* Rose et al., 2006). Another prosecutor tried to explain an ethnic minority victim’s allegedly excessive reaction by explaining to the judges that:

I think we should take into account that we may all have specific ways of reacting to crime, and the victim is reacting according to her experiences. (Field notes 2010)

However, this explanation results in a delegitimization of “foreign” emotions, and the power to define appropriate behavior is exercised with emotions as a cultural “weapon.” Abu-Lughod and Lutz (2009) similarly argue that emotions are used as an “idiom for communicating social conflict, and the ideal or deviant person,” and thus function as socially contested evaluations of the world. Accordingly, the feelings of majority Danish people are normalized, whereas those of ethnic minorities are deemed untrustworthy, which produces hierarchies of feelings and emotional domination (*cf.* Bonilla-Silva, 2019). The rejection of trustworthiness based on identities such as race or gender, therefore, is not merely an issue of contestation at a local level, but functions as a structural exclusion, giving rise to systematic injustices (Ahmed, 2004; Fricker, 2007). In light of this, the prosecutor seems to explain legal injustice through the coupling of “culture” and emotion, entailing that this injustice is stabilized and attributed to another ethnic group and foreign culture, rather than recognizing that the courtroom and legal system are themselves co-producers of “ethnicity” as well as structural inequality. The two victims’ emotional reactions might as well have been interpreted as a sign that they precisely perceive this institutional deligitimation, confirmed by one of the lay judges’ dismissive reactions.

## Trusted emotions: concluding discussion

It has been pointed out that legal practitioners may misinterpret communication with ethnic minorities, both in terms of different ways of explaining themselves, and different ways of expressing emotions (e.g., Gotaas, 2000; Shannon and Törnqvist, 2008). The behavioral codes may seem obvious to many legal professionals, and are therefore concealed from both themselves and the people who cannot decode the majority norms of language and behavior. The court’s understanding of emotions such as anxiety, anger, or grief shows the legal practitioners’ preoccupation with the victim, but this understanding is limited to certain accepted types of emotions, body language and social relations, which are further linked to the severity and situation of the case. As mentioned in the introduction, the Danish Institute for Human Rights is in the process of publishing a report which shows that victims from ethnic minority backgrounds more often experience that the defendant(s) are acquitted. As a quantitative study, it cannot explain why, but precisely because acquittals occur most often in the “ordinary” cases of violence, and not in rape cases or serious cases of violence, it seems fair to highlight the link that many legal professionals themselves make between trustworthiness and an incredibly strong emotional reaction in the courtroom in less serious cases.

While legal professionals may use empathy to understand different expressions of emotions, motivations, credibility and so on, this empathetic approach is challenged by unexpected or “matter out of place” emotions (Bladini et al., 2023). This suggests that “empathy” is neither a general nor a neutral entity. The analytical framework set out in this article has allowed me to go beyond issues of the (in)ability to empathize, and to explore the cultural assumptions that enhance or hamper this “empathy.” I argued that the legal expectations put on victims to be “calm and quiet” constitutes a specific and pervasive cultural model or schema that affects their credibility. Social judgments thus involve a process of categorization, which prompts legal professionals to translate information about people and events according to prior experiences, knowledge structures, and values that reach far beyond the courtroom and the legal context and rely on broader systems of knowledge. Being “calm and quiet” is an expression that similarly appears in other Danish institutional contexts such as schools (Gilliam, 2014), and in Danish popular culture such as music, literature, and film.^4^ When prosecutors and judges evaluate victims’ credibility, this assessment should be understood in light of both the legal context *and* broader semantic representations of emotions, i.e., how our societies construct emotions, and the ways in which people think about emotions (Anderson, 2011).

However, not all victims need to be equally “calm and quiet,” since this cultural expectation is mediated by gendered, majority perceptions of behavior. Intersectionality theory refines the idea of cultural models by focusing on identity categories such as gender, race, class, age etc., revealing how expectations regarding victims’ emotional reactions are also shaped by social identities constructed in specific locations and contexts (Yuval-Davis, 2006). This approach highlights the ways in which notions of own and others’ (emotional) cultures within the courtroom and beyond are intertwined with racism and discriminatory practices against immigrants in Denmark. By focusing on emotional behavior rather than social structures and power relations, legal professionals consolidate “culture” as being monolithic, static, in short, culture as essence (*cf.* van Oorschot, 2023). My own discussion of legal professionals’ cultural preconceptions similarly risks portraying “calm and quiet” as imperative cultural model. However, the analytical approach to knowledge structures does not imply that all people in Denmark think, feel or behave similarly, or that it is impossible to desist or raise awareness about specific cultural understandings. It might seem tempting to suggest that judges should raise their awareness about “other cultures,” for instance through cultural sensitivity training. However, as discussed by Rossmanith (2023), this kind of training may lead to overconfident judges who feel they can “read” people from other cultures or social groups, based on generalized knowledge unintentionally reproducing stereotypes and biases. Rather than offering judges awareness training about *other* peoples’ cultures, then, it might be more fruitful to suggest raising awareness about their own implicit categorizations as well as the cultural assumptions they are based on.

## Data Availability

The datasets presented in this article are not readily available for sharing. Requests to access the datasets should be directed to louise.victoria.johansen@jur.ku.dk.

## Footnotes

^1^This article thus focuses on a defined aspect of “credibility.” Credibility assessments in court of course rely on a number of other factors than emotions, such as criminal evidence, statements, the relative trustworthiness of the parties, witnesses etc., a point that will be elaborated in the section about the Danish criminal justice system.

^2^I use the name “criminal justice actor” in this paper to address a broader range of professionals such as the police and probation officers, whereas ‘legal professionals’ includes only people with formal legal training such as judges, prosecutors, etc.

^3^Since exaggerations seem to challenge the Danish feeling rule the most, I have prioritized these examples in the following, although I do analyse ‘downplayed’ emotional reactions elsewhere (Johansen et al., 2023).

^4^For instance, the Danish folk and rock singer Kim Larsen wrote the song “calm and quiet,” there is a Danish children’s short movie, and a cartoon with the same title, just to mention some examples.
